# Cross-cultural adaptation and validation of Behçet's disease quality of life questionnaire

**DOI:** 10.1186/1471-2288-11-52

**Published:** 2011-04-20

**Authors:** Zahi Touma, Lilian Ghandour, Abla Sibai, Houry Puzantian, Ayad Hamdan, Omar Hamdan, Jeanine Menassa, Imad Uthman, Thurayya Arayssi

**Affiliations:** 1 University of Toronto, Centre for Prognosis Studies in the Rheumatic Diseases, Toronto, Ontario, Canada; 2 Department of Epidemiology and Population Health, American University of Beirut, Beirut, Lebanon; 3 School of Nursing, University of Pennsylvania, Philadelphia, Pennsylvania, USA; 4 Harvard Medical School, Beth Israel Deaconess Medical Center, Boston, Massachusetts, USA; 5 Department of Internal Medicine, Division of Rheumatology, American University of Beirut, Beirut, Lebanon; 6 Weill Cornell Medical College-Qatar, Education City, Doha Qatar

**Keywords:** Behçet's disease, Quality of life, Arabic version of BD-QoL, Cross-cultural adaptation, Validity and reliability

## Abstract

**Background:**

Currently, there is one Behçet's disease (BD) specific self reporting questionnaire developed and published in the literature, The Leeds BD-quality of life (QoL). We conducted a cross-cultural adaptation and validation of the Arabic version of the Leeds BD-QoL

**Methods:**

A cross-sectional study was conducted among 41 consecutive patients attending rheumatology clinics at the American University of Beirut Medical Center between June and December 2007. The BD-QoL questionnaire, the Katz Index of Activities of Daily Living (ADL) and the Lawton Instrumental Activities of Daily Living (IADL) questionnaires were co-administered during the same visit, and severity scores were calculated. Cross-cultural adaptation of BD-QoL was performed using forward and backward translations of the original questionnaire. Internal consistency and test-retest reliability of the final version were determined. Exploratory Factor Analysis (EFA) was used to assess the dimensionality of the scale items. External construct validity was examined by correlating Arabic BD-QoL with the severity score, ADL and IADL.

**Results:**

The 30 items of the adapted Arabic BD-QoL showed a high internal consistency (KR-20 coefficient 0.89) and test-retest reliability (Spearman's test 0.91). The convergence of all 30 items suggests that the 30-item adapted Arabic BD-QoL scale is unidimensional. BD-QoL did not correlate with any of the patients' demographics. Still, it was positively correlated with patient severity score (r 0.4, p 0.02), and IADL (but not ADL).

**Conclusions:**

This cross-cultural adaptation has produced an Arabic BD-QoL questionnaire that is now available for use in clinical settings and in research studies, among Arabic speaking patients.

## Background

Behçet's disease (BD) is a multisystem inflammatory disorder of unclear etiology characterized by recurrent oral and genital ulcers, skin lesions and uveitis. Other manifestations include arthritis, a positive pathergy test, thrombophlebitis, central nervous system disease and gastrointestinal ulcerations [1-4]. Patients with BD suffer a variety of activity limitations (disability) and restriction of participation in many areas of life (handicap) [5].

Health related quality of life HRQoL has become an important outcome measure in the assessment of chronic disabling conditions, including BD [5,6]. While disease generic questionnaires as the Medical Outcomes Study Short-Form (SF-36) and others are used for patients with musculoskeletal diseases, it has been advocated that disease-specific HRQoL questionnaires are better suited than generic instruments for rheumatic diseases, as they might be more responsive to clinical change, and are appropriate to evaluate specific therapeutic interventions [7-12]. Currently, there is one BD-specific self reporting questionnaire developed and published in the literature, the Leeds BD-QoL available in the English and Korean languages [13].

The aim of the present study is to conduct a cross-cultural adaptation and validation of an Arabic version of the BD-QoL and produce a reliable and valid Arabic version of the instrument.

## Methods

### Patient Recruitment and Assessment

Forty one patients satisfying the International Study Group (ISG) criteria for diagnosis of BD and attending the rheumatology clinics at the American University of Beirut-Medical Center (AUB-MC) between June and December 2007 were enrolled [14]. Patients enrolled in this study did not have psychiatric illness or psychiatric treatments that would affect their ability to complete the questionnaire. The Leeds BD-QoL, the Katz Index of Activities of Daily Living (ADL) and the Lawton Instrumental Activities of Daily Living (IADL) questionnaires were self-administered to the patients on the day of the visit day [5,15,16]. A complete history and physical exam were also performed at the visit. Symptoms at the time of the study visit were recorded and those suffered over the course of the disease were derived from the Behçet's Disease Database at AUB-MC [17]. The study was approved by the Institutional Review Board and informed consent was obtained from all patients.

### Clinical Severity Score

Severity score was calculated as the sum of 1 point for each mild symptom (oral aphthosis, genital ulcers, arthralgia and typical skin lesions such as erythema nodosum, papulopustular lesions and folliculitis), 2 points for each moderate symptom (arthritis, deep vein thrombosis of the legs, anterior uveitis and gastrointestinal involvement) and 3 points for each severe symptom (posterior/panuveitis, retinal vasculitis, arterial thrombosis, neuro-Behçet's and bowel perforation). Patients were categorized into three groups according to their score: severe (≥7), moderate (4- 6) and mild group (<4) [18].

### Outcome Measures Used to Assess Quality of Life

***Leeds BD-QoL ***is a composed of 30 items, answered true (1) or false (0), yielding a score ranging from 0 to 30 [5]. Prior to the study, the developers of the Leeds BD-QoL were contacted and the permission to use the scale was granted.

***Katz ADL***: the Katz Index of Activities of Daily Living (ADL) scale is a 6-item tool used to assess functional dependency status of individuals [15]. ADL disability was defined as needing help with one or more of the activities.

***Lawton IADL: ***the Lawton Instrumental Activities of Daily Living (IADL) scale captures a different dimension of disability than Katz ADL scale. IADL disability was defined as having difficulty in one or more of activities [16].

### Cross-Cultural Adaptation

Guillemin and colleagues suggest five different examples of cross-cultural adaptation; the last scenario is the application of the questionnaire in a different culture, language and country [19]. Cross-cultural adaptation aims to reach the equivalence between the original source (in the UK) and target version (in Lebanon) of the questionnaire. More importantly all items must be adapted culturally to maintain the content validity of the scale at a conceptual level across different cultures [20,21]. We followed the five steps described by Guillemin and colleagues and Beaton and colleagues, intended for a questionnaire of self-report-health status measures, as follows [19,20].

***Stage I***: initial translation; a bilingual translator whose mother tongue is Arabic generated an independent translation in Arabic, referred to as T1. The translator produced a written report of the completed translation. The translator was not aware of the concept being quantified and had no medical or clinical background about BD.

***Stage II***: synthesis of the translation; three authors (Z.T., A.S., T.A.) synthesized the results of the translation and agreed on a common modified Arabic version, referred to as T2. Their comments were mostly concerning the wording of the Arabic questionnaire and whenever appropriate modifications were made.

***Stage III***: back translation (BT); two English translators, working from the T2 version, translated back the Arabic version of the BD-QoL into English. Both translators were totally blind to the original version, unaware of the concepts explored, and had no medical background. The first translator was a certified translator and the second was an MSc student, both native speakers of Arabic and proficient in English. Translators produced a written report of the back translations (BT1 and BT2) that they completed. Three authors (Z.T., A.S., and T.A.) synthesized one English version (BT12). BT12 was then cross-examined with the original English version and discrepancies corrected. This version was later tested on 5 healthy individuals who were native speakers of Arabic and fluent in English, reviewing BT12 version and providing critical feedback on the vocabulary. This was to ensure different aspects of cross-cultural validity.

***Stage IV***: expert committee; a committee was set up with the aim to cross-examine all versions against the original BD-QoL questionnaire and produce the "prefinal version" of the Arabic BD-QoL. This Committee was compromised of two rheumatologists, experts in BD (T.A. and Z.T.), an epidemiologist (A.S.), translators and a clinical research nurse coordinator (H.P.). Each item was discussed and evaluated from all the viewpoints of the different professionals in the committee. Their comments were mostly concerning the wording of the questionnaire and where appropriate, changes were made in accordance with the suggestions. This resulted in a pre-final version of the Arabic questionnaire that was tested on 3 healthy individuals and 3 patients with medical illnesses other than BD. All 6 participants completed the questionnaire within 5 minutes, and reported that all items, except for one - item 21, were simple and easy to understand.

***Stage V***: test of the pre-final version; 5 patients with BD and 6 patients with rheumatoid arthritis completed the Arabic pre-final version of the BD-QoL questionnaire. The objective of this stage is to ensure that the adapted version retains the adequacy of content, clarity of wording and usefulness. All patients completed the questionnaire and highlighted that the Arabic BD-QoL was easy to understand and BD patients confirmed its adequacy in addressing the QoL related to their disease. The final version of the Arabic BD-QoL was then ready for further statistical evaluation of its psychometric properties, its reliability and validity (Table 1).

**Table 1:** Percent responses to BD-QoL as recorded by the 41 patients

Questions	n	%	Questions	n	%
**Q1**	**12**	**29**	**Q16**	**17**	**41**

**Q2**	**7**	**17**	**Q17**	**19**	**46**

**Q3**	**14**	**34**	**Q18**	**12**	**29**

**Q4**	**13**	**31**	**Q19**	**16**	**39**

**Q5**	**11**	**27**	**Q20**	**16**	**39**

**Q6**	**22**	**54**	**Q21**	**19**	**46**

**Q7**	**12**	**29**	**Q22**	**14**	**34**

**Q8**	**10**	**24**	**Q23**	**5**	**12**

**Q9**	**6**	**15**	**Q24**	**21**	**51**

**Q10**	**10**	**24**	**Q25**	**11**	**27**

**Q11**	**7**	**17**	**Q26**	**7**	**17**

**Q12**	**11**	**27**	**Q27**	**6**	**15**

**Q13**	**10**	**24**	**Q28**	**18**	**44**

**Q14**	**11**	**27**	**Q29**	**7**	**17**

**Q15**	**22**	**54**	**Q30**	**8**	**19**

### Analysis

***Descriptive statistics ***were used to characterize the patients' socio-demographic and clinical manifestations, severity scores and BD-QoL. All analyses were undertaken using Stata 10.0. A two-sample independent t-test was conducted to compare means across binary variables (e.g., BD-QoL scores by age), and one-way ANOVA was used to compare means across categorical variables.

#### Test-retest reliability was determined among 10 patients at 1 week intervals

The Spearman's test was applied to determine the correlation coefficient. A correlation coefficient of at least 0.7 is considered acceptable and anything over 0.8 is considered to be high [22,23].

***Internal consistency ***was assessed using Kuder-Richardson-20 (KR-20) coefficient of the set of 30 dichotomous items [23,24]. For research and group level comparisons, KR-20 of 0.80 or higher is considered acceptable and KR-20 > 0.9-0.95 may represent "redundancy" in the scale and, thus allow further item reduction [22-24].

#### Construct validity

Exploratory Factor Analysis (EFA) was used to assess the dimensionality of the items in the scale, or its factor structure. Prior to running the EFA, the relative frequency distributions of all 30 items were carefully explored. The assessment scale was binary (true, not true), and thus the correlations computed by the statistical software were tetrachoric correlations. Principal Components Analysis (PCA) was used as the extraction method. To empirically examine the number of factors, a number of measures were examined: eigenvalues, a scree plot, and item factor loadings. An eigenvalue greater than one is typically used to reflect the number of underlying factors; however, this criterion alone does not suffice as it tends to overestimate the number of factors to be extracted. The scree plot is a visual depiction of the eigenvalues; the number of data points above the "elbow" indicates the appropriate number of factors to be extracted [25].

The external nomological construct validity was examined by correlating the score of the Arabic version of BD-QoL with the severity scores (using Pearson's correlation and linear regression), as well as ADL and IADL (using two-sample independent t-test). We further studied the correlation between BD-QoL and the patients' demographics. Using a linear regression model, and while controlling for IADL and ADL, we examined the ability of BD severity (as determined by severity index) to predict BD-QoL.

## Results

### Patients' Demographics

The total sample (n = 41) included 24 males and 17 females. The mean age at the time of the study was 34 ± 11 years. The characteristics of the patient in represented in table 2. All patients were Lebanese whose mother tongue is Arabic. Patients displayed a spectrum of clinical manifestations during the progression of their disease, and at the time of the study visit.

**Table 2:** Socio-demographic characteristics and clinical manifestations of 41 patients with BD

	Number of patients	Percentage of patients
**Socio-demographics**		

**Gender**		

Male	24	59

Female	17	41

**Education**		

Illiterate	1	2.4

Read and Write	1	2.4

Primary	4	9.8

Intermediate	8	19.5

Secondary	7	17.1

Technical	5	12.2

University	15	36.6

**Marital Status**		

Single	20	48.8

Married	20	48.8

Divorced	1	2.4

**Employment**		

Unemployed	20	51.2

Employed	19	48.8

**Clinical Manifestations**		

Oral ulcers	41	100.0

Genital Ulcer	30	73.2

Skin manifestations	41	100.0

Eye Disease	29	70.7

Blindness	2	4.9

Vascular involvement	14	34.1

Neurologic involvement	27	65.9

Athralgia/arthritis	29	70.7

Epidedimitis	3	7.3

### Severity Score

The mean of the severity score was 9 ± 3.41 and was statistically higher in males (10.08) compared to females (7.47) (*p-value*: 0.01). The severity score was not related to any other variable (age, educational level, living arrangement, employment or marital status of the patients).

### BD-QoL

The mean BD-QoL was 9.12 (SD 6.69, and 95% CI 7.01-11.12); the percent distribution of the responses to each item are shown in table 2. The BD-QoL scores were higher in males (10.04, SD 6.89) compared to females (7.82, SD 6.38) but not statistically significant. The mean of BD-QoL scores were significantly higher in the group of patients older than 35 years (7.10, SD 4.91) compared to those aged 18-35 years (11.47, SD 7.80) [*p-value*: 0.03]. Despite being statistically significant, these results should be interpreted with caution given the width of CI. The way we grouped our patients could have resulted in this statistically significant result. There was a trend for higher BD-QoL scores in the group with lower education but the finding was not statistically significant (p = 0.11). The BD-QoL scores were not related to the patients' marital or employment status (Table 3)

**Table 3:** Comparing BD-QoL scores by demographics for 41 patients

	Mean	SD	95% CI	*p *value
**Sex**				
Male (n = 24)	10.04	6.89	7.13, 12.95	0.30
Female (n = 17)	7.82	6.83	4.54, 11.10	

**Age**				
18-35 years (n = 22)	7.10	4.91	4.92, 9.26	0.03
>35 years (n = 19)	11.47	7.80	7.72, 15.23	

**Marital status**				
Single (n = 20)	9.45	5.62	6.81, 12.08	0.76
Ever married (n = 21)	8.81	7.71	5.30, 2.32	

Education*				
Low (n = 6)	12.33	10.00	4.32, 20.34	0.11
Intermediate (n = 20)	10.05	6.50	7.23, 12.87	
High (n = 15)	6.6	4.81	4.17, 9.03	

**Employment**				
Student (n = 6)	4.33	2.94	1.98, 6.68	0.15
Home maker (n = 7)	8.14	8.10	2.15, 14.13	
Part time (n = 4)	11.75	9.11	2.84, 20.66	
Full time (n = 18)	9.10	5.42	6.57, 15.62	
Disabled (n = 5)	14.2	8.76	6.52, 7.68	

Number of household members**				
0-5 (n = 22)	7.95	5.34	5.59, 10.32	0.23
6-9 (n = 19)	10.47	7.92	6.66, 14.30	

* Low (illiterate, read and write, and primary), Intermediate (intermediate, secondary, and technical), and high (university)

** Number of household members dichotomized as mean or below, and above mean

### Activities of Daily Living (ADL/IADL)

Around 27% of the patients had difficulty in at least one ADL or IADL items and 27% patients had difficulty in at least one IADL items (17% of the patients reporting both).

### Test-retest reliability and internal consistency of the final Arabic version

The 30 items of the final adapted Arabic BD-QoL showed a high internal consistency (KR-20 coefficient 0.89) and a high test-retest reliability (Spearman's test; 0.91, p < 0.01).

### Validity

#### Content Validity

In stage IV of the cross-cultural adaptation, the construction of the sentences in the translated version of BD-QoL proved to be the major source of debate among the committee members. In some cases items were rephrased to make them comprehensible, still maintaining the original meaning. We kept the language in the Arabic version informal and simple, following the English version, although in some cases the wording was slightly modified to better suit the Arabic style.

Few expressions proved problematic: in particular Q5, Q7, Q9, Q10, Q15, and Q21, for the forward translators, consequently causing the back translation to be discordant with the original version. The Committee agreed on the following modifications of the final Arabic version. For Q5 we added "regular things" in the Arabic version referring to "daily activities", and for Q7 we added "daily life" to the Arabic version instead of "life". Q9 was simplified in the Arabic version although keeping the original meaning of the English version, for Q10 "stressful" was substituted by "disturbed" in the Arabic version, and for Q15 "rely" was substituted by "trust" in the Arabic version. Q21 was unclear for patients and we added to the sentence (because of mouth ulceration) in the Arabic version. In stage V patients confirmed that the Arabic version of BD-QoL was easy to understand and useful. A final version of the Arabic BD-QoL was generated and is available upon request.

#### Internal Construct Validity: Convergence of items

Upon examining the PCA results of EFA, 10 components had a value greater than 1; the first component however had an eigenvalue of 8.77 followed by 2.38 for the second, 2.09 for the third, 1.90 for the fourth, with the remaining 6 components ranging from 1.07-1.68. The first component accounted for 29% of the variance, followed by 4-8% for each of the remaining 9 components. While the eigenvalues suggest the extraction of 10 factors, the eigenvalue rule more often than not leads to an overestimation of the number of underlying latent factors.

**Figure 1:**
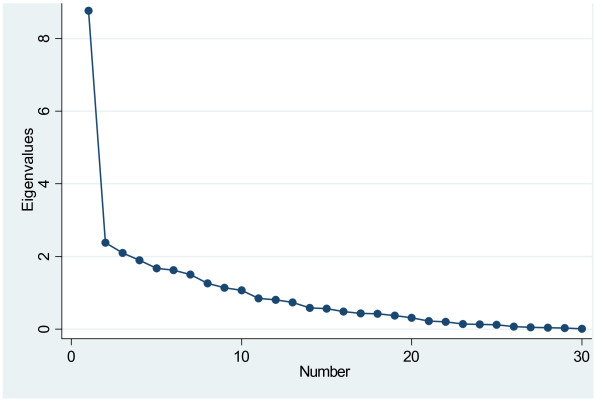
**Screeplot of 30-item adapted Arabic BD-QoL**.

The scree plot, suggested that a 1-factor solution is optimal (Figure 1); in other words, the 30-item scale is unidimensional. The ratio of the first to second eigenvalue in our sample was almost four (3.7), which is further evidence for unidimensionality of the scale.

Upon examining all measures for extracting factors, the results suggest that the 30-item scale is unidimensional which means that the scale is measuring the same thing (attribute) (Figure 1). The unidimensionality of the adapted Arabic BD- QoL version is further evidence to the convergence of all 30 items and is an indication of its construct validity [25].

#### External Nomological Validity

BD-QoL did not correlate with any of the patients' demographics. There was a statistically significant positively moderate correlation between the severity score and BD-QoL (r 0.4, *p-value *0.02). A one-unit increase in the severity score was shown to predict a 0.7 unit increase in the BD-QoL total score (β = 0.71, *p-value *= 0.02, 95% CI 0.11-1.30).

BD-QoL was also independently related to IADL (but not ADL) whereby the mean BD-QoL severity scores were statistically significantly higher in those with any IADL (10.81, 95% CI 8.39-13.23 vs. 8.33, 95% CI 7.16-9.50 (*p-value*: 0.03). This could be potentially explained by the fact that several of the IADL questions overlap with BD-QoL items, in contrast to ADL, which is used to assess the functional status of physically dependent individuals.

Severity of BD was predicted by BD-QoL controlling for the presence of any IADL (β: 0.71, 95% CI 0.10-1.30 *p-value *= 0.02), but not while controlling for any ADL item (β: 0.53, 95% CI -0.08-1.14, *p-value *= 0.08).

## Discussions

The evidence supporting the benefits of using a disease-specific questionnaire to assess quality of life in rheumatology including BD in English-speaking countries call for an extension of their availability to other linguistic groups. This becomes particularly important since disease-specific quality of life measures may evaluate the impact of the disease on areas that are not adequately addressed in routine clinical practice or in generic questionnaires that may lack one or more domains pertinent to BD patients (e.g., problems with talking and eating for patients with mouth ulceration, effect on relationships).

The present paper described the cross-cultural adaptation and validation of the Arabic version BD-QoL. The multidisciplinary nature of our expert committee was crucial in reaching an appropriate version, examining the work from different points of view. The BD-QoL was translated from English into Arabic using a rigorous methodology; words were replaced, as necessary, to render BD-QoL items more valid in the Arabic language within our cultural context [20]. Feedback expressed by the patients on each item of the questionnaire in stage V of the process provided an indispensable perspective that ensured the simplicity and comprehensibility of the Arabic version. For most patients, it took less than 10 minutes to complete the questionnaire. Furthermore, the Arabic version of the BD-QoL was shown to be unidimensional, highly reliable and of adequate construct validity.

Patients in this study presented a relatively high severity score of their disease. The major impact of the disease was found in items 6, 15 and 24 that addressed the patient's ability to stand for long, his perception about the future of his life and disease, and his own worries about holding others back, respectively. This also illustrates how BD affects several domains of a patient's life. Less impact was observed for items 9, 23 and 27, which assess the patient's looks, their feeling of uselessness due to the disease and their contact with people, respectively. This could be related to the ability of the patient to accept, cope and live with the disease. In our study we showed a moderate correlation between the BD-QoL and the severity score. Indeed, we expected this result especially that previous studies in BD and other rheumatic diseases have shown that HRQoL do not always correlate strongly (>0.7) with disease severity. Furthermore, HRQoL assessment is considered as an independent outcome measure in the assessment of rheumatic diseases. The 'Outcome Measures in Rheumatology' (OMERACT) IV, recommended for both randomized clinical trials and longitudinal observational studies, including HRQoL as an outcome measure among others [6]. Similar to our results, a study on HRQoL in 41 BD patients showed a low to moderate correlation with disease activity [26]. More recently, Ertam et al., showed that HRQoL in 195 BD patients is impaired and related with disease severity in BD [10].

The mean of BD-QoL scores were higher in males compared to female but not statistically different; whether the higher severity scores contributed to higher BD-QoL scores needs to be addressed in future studies. BD disease activity is usually more severe among younger patients and one would expect a lower HRQoL in this group. However, in our study the older group (age > 35 years) as compared to the younger group (age < 35 years) had lower HRQoL with a higher score on the BD-QoL questionnaire. Probably a longer disease duration is a factor implicated in the worsening of HRQoL with the potential damage accrue that might occur with BD. All these research questions need to be evaluated and addressed in multi-center studies with patients of different ethnicities and larger sample size.

Upon comparing our results with the reference values from a Korean study that administered the BD-QoL questionnaire consecutively to 201 patients, BD-QoL scores in Koreans were lower. Moreover, BD-QoL scores in their sample (predominantly female, 61%) were statistically higher in females, a finding that was possibly attributed to the higher rate of depression in women.

Despite its offsetting strengths, the present study and its findings need to be interpreted in light of a few limitations. First, the sample size was small, particularly for the exploratory factor analysis (EFA). Several different recommendations have been made for the optimum sample size for an EFA pertaining to either the absolute sample size or the subject-to-variable ratios. Our sample of 41 patients falls below the recommended levels, yet was sufficient enough to exhibit sufficient power and detect expected and statistically significant associations between BD-QoL and other measures. Moreover, the high internal consistency of the 30 items speaks to the unidimensionality of the scale (as evidenced by the EFA).

## Conclusions

In summary, there is evidence that BD-QoL is accepted by BD patients and it correlated with the severity score of the disease. An Arabic version Behçet's disease quality of life is now available for testing and use in clinical settings and research studies that assess the quality of life in Arabic speaking patients. The majority of the BD patients are from the areas surrounding the old Silk Road in the Middle East and in Central Asia. We believe that the use of the Arabic version of BD-QoL questionnaire together with the English, Korean and other potential versions of BD-QoL would allow comparison of cross-national findings.

### What is already known on this subject?

Currently, there is one BD-specific self reporting questionnaire developed and published in the literature, the Leeds BD-QoL along with its Korean version.

### What does this study add?

Behçet's Disease is more common in the Middle East and Asia. This cross-cultural adaptation and validation has produced an Arabic BD-QoL questionnaire that can be used in clinical and in research studies in these geographic areas. The Arabic version of the Leeds BD-QoL questionnaire is reliable and valid.

## Conflict of interest statement

The authors declare that they have no competing interests.

## Authors' contributions

All authors participated in the study design, analysis and interpretation of data, and preparation of the manuscript. All authors read and approved the final manuscript.

## Pre-publication history

The pre-publication history for this paper can be accessed here:

http://www.biomedcentral.com/1471-2288/11/52/prepub
